# Clinical Outcomes and Healthcare Resource Utilization Results Among Hospitalized Adults with Cystic Fibrosis Treated with Ceftolozane/Tazobactam: A SPECTRA Real-World Subgroup Analysis

**DOI:** 10.3390/antibiotics15080715

**Published:** 2026-07-23

**Authors:** Emre Yucel, Alex Soriano, Florian Thalhammer, Stefan Kluge, Mike Allen, Jessica Levy, Huina Yang, Sunny Kaul

**Affiliations:** 1 Merck & Co., Inc., 351 North Sumneytown Pike, P.O. Box 1000, North Wales, PA 19454, USA; 2 Department of Infectious Diseases, Hospital Clinic, Carrer de Villarroel, 170, L’Eixample, 08036 Barcelona, Spain; 3 Department of Urology, Medical University of Vienna, Spitalgasse 23, 1090 Vienna, Austria; 4 Department of Intensive Care Medicine, University Medical Center Hamburg-Eppendorf, 20251 Hamburg, Germany; 5 MSD (UK) Limited, 120 Moorgate, London EC2M 6UR, UK; 6 Department of Laboratory Medicine, Tan Tock Seng Hospital, 11 Jln Tan Tock Seng, Singapore 308433, Singapore; 7 Royal Brompton & Harefield NHS Foundation Trust, Britten St, London SW3 6PY, UK

**Keywords:** ceftolozane/tazobactam, cystic fibrosis, real-world, clinical outcomes, healthcare resource use, antimicrobial stewardship, SPECTRA

## Abstract

**Background:** Patients with cystic fibrosis (CF) experience recurrent infections requiring repeated antimicrobial therapy, leading to high rates of multidrug-resistant (MDR) *Pseudomonas aeruginosa* (*P. aeruginosa*) and contributing substantially to disease-related mortality. Ceftolozane/tazobactam (C/T) is an innovative therapy used to treat resistant Gram-negative infections, with enhanced activity against *P. aeruginosa* infections. **Methods:** The Study of Prescribing patterns and Effectiveness of Ceftolozane/Tazobactam Real-world Analysis (SPECTRA) was a multicenter, retrospective real-world study of hospitalized adults who received ≥48 h of C/T across seven countries. Medical record data were collected for up to 6 months before treatment and 30 days after the final C/T dose (or death). This subgroup analysis describes clinical outcomes and healthcare resource utilization (HCRU) among hospitalized adults with CF who received C/T for an index infection. **Results:** Of 64 patients with CF, 53.1% had MDR *P. aeruginosa*. Clinical success was achieved in 78.1% of patients, with highest and lowest success rates in patients treated with C/T as second (100%) and sixth or later (40.0%) treatment. Microbial eradication was reported in 10.9% of patients. In-hospital mortality occurred in 14.1% of patients, with the lowest percentage in patients treated with C/T as first or second line treatment (0.0%) and the highest in patients treated with C/T sixth or later (40.0%). Median LOS was 31.5 days. Readmission (30-day all-cause) occurred in 10.9% of patients. **Conclusions:** In this SPECTRA subgroup analysis, C/T was associated with favorable real-world clinical outcomes among hospitalized adults with CF treated for an index infection. Interpretation is limited by the retrospective design, heterogeneous infection indications/sites, and limited interpretability of microbiologic endpoints in CF.

## 1. Introduction

Cystic fibrosis (CF), a condition which affects more than 70,000 people globally, is a multi-organ genetic disease caused by defects in the CF transmembrane conductance regulator gene, which results in ion channel dysfunction throughout the body [1,2]. As a result, patients with CF are prone to the production of thick mucus in the lungs, which affects airway clearance, causes progressive lung disease and accounts for the majority of the morbidity and mortality in CF patients [1,2,3]. Due to the overproduction of mucus, patients with CF often experience repeated bacterial infections, causing damage to bronchial walls and leading to permanent loss of lung function [1,2,3].

Throughout their lifetime, patients with CF are frequently treated with antibiotics to manage these infections, with some receiving long-term or preventative antibiotic treatment [2,4]. Repeated exposure to antibiotics increases the risk of hypersensitivity reactions and development of antimicrobial resistance (AMR), leading to an increase in multidrug-resistant (MDR) pathogens in these patients [1,5]. Specifically, *Pseudomonas aeruginosa* (*P. aeruginosa*) is one of the most common pathogens associated with lung infections in CF, present in up to 70% of adults with CF [1,6]. The eradication of *P. aeruginosa* is particularly challenging to healthcare providers due to its ability to form biofilms, causing AMR, rapid pulmonary decline and contributing to early death [1,6].

The rising AMR and increasing difficulty in pathogen eradication, coupled with hypersensitivity reactions resulting from recurrent antimicrobial use in patients with CF, mean that effective therapeutic strategies are becoming increasingly limited [2]. Innovative therapies such as ceftolozane/tazobactam (C/T) combines a cephalosporin with targeted activity against *P. aeruginosa* and a β-lactamase inhibitor designed to extend activity against resistant Gram-negative organisms. This combination addresses a growing clinical need in settings where resistance reduces the utility of standard therapies. Its antimicrobial profile has supported its use in complex infections where effective treatment options may otherwise be limited [7].

While randomized controlled trials (RCTs) remain the gold standard and establish treatment efficacy under controlled conditions, they often exclude patients with comorbidities, such as patients with CF [8]. As a result, trial findings may not fully capture outcomes in routine practice, where patients frequently present with greater clinical complexity and comorbidity and real-world evidence is required to complement findings from RCTs to improve understanding of treatment effectiveness over a wider range of patient and infection types. Observational data derived from real-world clinical settings provide an important complement to trial evidence by reflecting treatment use and outcomes across a broader spectrum of patients. Such data are increasingly considered in the evaluation of therapeutic effectiveness in clinical and policy decision-making [9].

The Study of Prescribing patterns and Effectiveness of Ceftolozane/Tazobactam Real-world Analysis (SPECTRA) study is a retrospective, multinational analysis designed to evaluate the use of ceftolozane/tazobactam in routine clinical practice. The study includes hospitalized patients treated across multiple countries and captures detailed information on treatment patterns, clinical outcomes, and microbiological findings as documented in medical records. Prior analyses have described outcomes in the overall cohort and in selected high-risk populations [10,11,12]. The present analysis focuses specifically on patients with cystic fibrosis, with the aim of characterizing outcomes and healthcare resource utilization within this subgroup.

## 2. Results

Index infection site/indication (e.g., respiratory, urinary, intra-abdominal) was not consistently captured for this CF subgroup; therefore, outcomes should be interpreted as descriptive results among hospitalized adults with CF treated with C/T for heterogeneous index infections.

### 2.1. Patient Characteristics

In total, 64 of the 617 (10.4%) patients included in the SPECTRA study had CF. Of those, 34 (53.1%) had MDR *P. aeruginosa*. The rank of C/T initiation was most commonly a second line treatment (*n* = 16), third line treatment (*n* = 11), first line treatment (*n* = 10) or sixth line treatment or more (*n* = 10). The majority of these patients were from the UK (*n* = 35, 54.7%), Australia (*n* = 12, 18.8%) and Spain (*n* = 11, 17.2%; Table S1). The most common comorbidities observed in patients with CF were chronic pulmonary disease (*n* = 45, 70.3%), exacerbation of chronic respiratory infection (*n* = 36, 56.3%), immunocompromised status (*n* = 36, 56.3%) and organ transplant (*n* = 33, 51.6%; Table S2). Where reported, most patients with CF had normal renal function (60.9%) and body mass index (BMI; 78.0%; Table S2). These comorbidities reflect underlying clinical history recorded in the medical record and do not necessarily represent the index infection for which C/T was administered.

### 2.2. Clinical Outcomes

**Figure 1:**
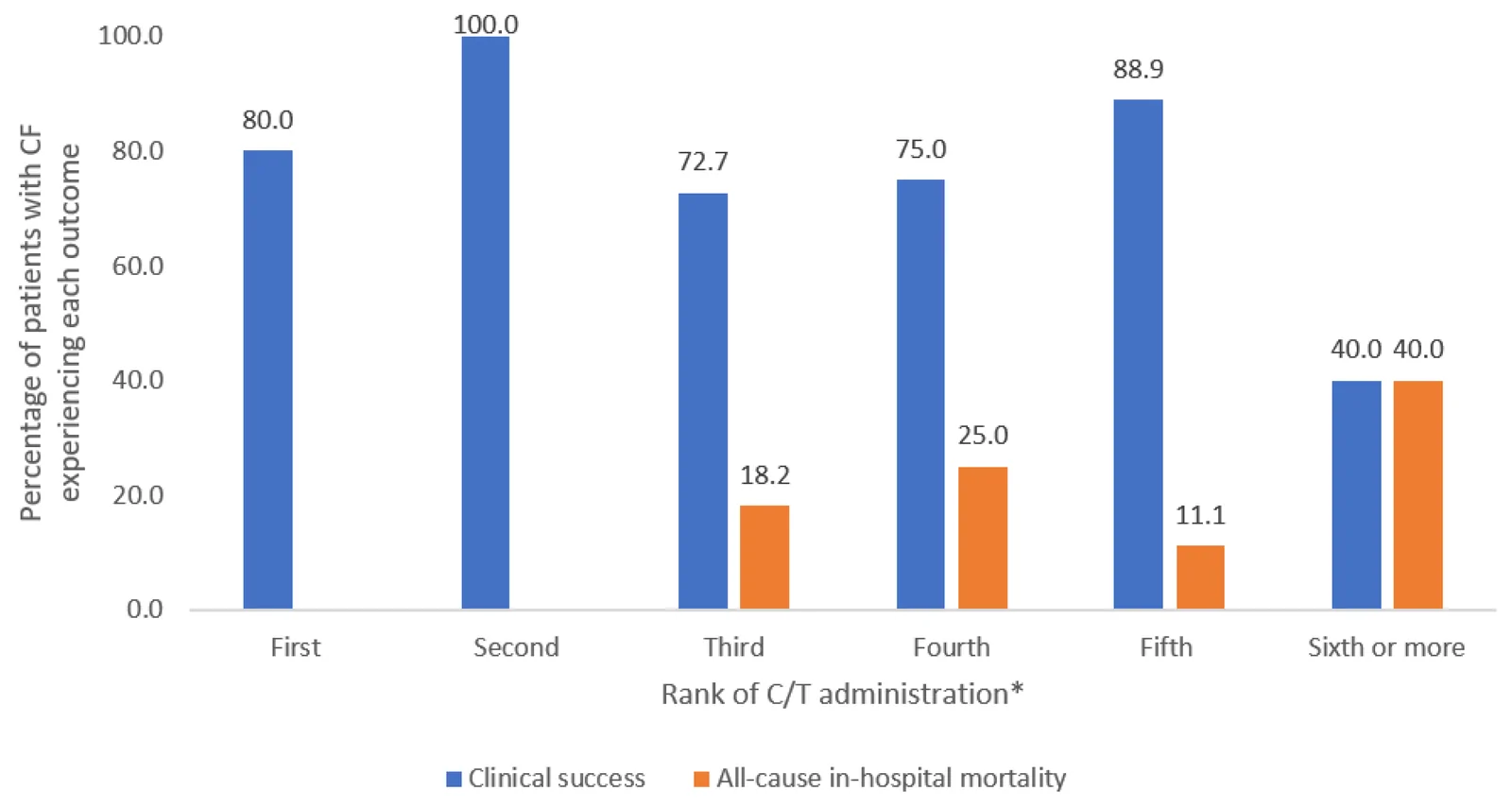
Clinical success and all-cause in-hospital mortality rates in patients with CF by rank of C/T initiation. CF: cystic fibrosis, C/T: ceftolozane/tazobactam. * Rank of C/T initiation was defined by chronological order of start dates of all anti-pseudomonal Gram-negative antibacterials given for the index infection.

Clinical success was recorded in 78.1% of patients with CF overall (95% CI: 66.0, 87.5; Table 1). Clinical success rates varied by rank of C/T initiation, with highest rates recorded in patients where C/T was given as second line treatment (100%; 95% CI: 79.4, 100.0), followed by fifth line treatment (88.9%; 95% CI: 51.8, 99.7), first line treatment (80.0%; 95% CI: 44.4, 97.5), fourth line treatment (75.0%; 95% CI: 34.9, 96.8), third line treatment (72.7%; 95% CI: 39.0, 94.0) and finally sixth line treatment or more (40.0%; 95% CI: 12.2, 73.8; Table 1; Figure 1).

Microbial eradication was reported in 10.9% of patients with CF overall (95% CI: 4.5, 21.2). When reporting by rank of C/T initiation, microbiological eradication was highest in patients who received C/T as first line treatment (20.0%; 95% CI: 2.5, 55.6) and lowest in patients who received C/T as fifth treatment (0.0%; 95% CI: not applicable [NA]; Table 1).

Overall, all-cause in-hospital mortality was observed in 14.1% of patients with CF (95% CI: 6.6, 25.0). All-cause in-hospital mortality was lowest in patients who received C/T as first or second line treatment (0.0%; 95% CI: NA) and highest in patients who received C/T as the sixth or later treatment (40.0%; 95% CI: 12.2, 73.8; Table 1; Figure 1). Similarly, infection-related in-hospital mortality was observed in 9.4% of patients with CF overall (95% CI: 3.5, 19.3). Infection-related in-hospital mortality was lowest in patients who received C/T as first or second line treatment (0.0%; 95% CI: NA) and highest in patients who received C/T as sixth or later treatment (30.0%; 95% CI: 6.7, 65.2; Table 1).

### 2.3. Healthcare Resource Utilization

**Figure 2:**
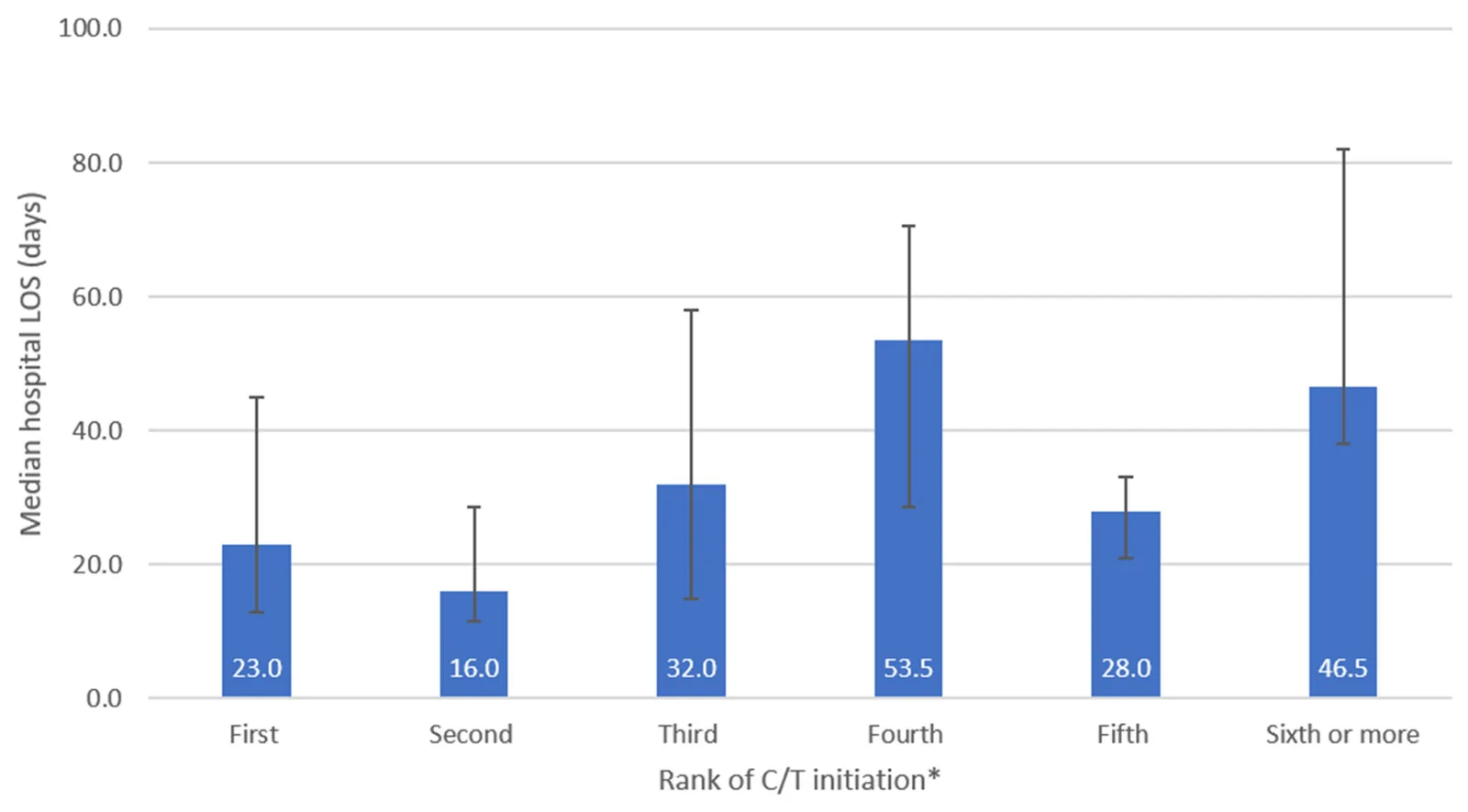
Median hospital LOS in patients with CF by rank of C/T initiation. CF: cystic fibrosis, C/T: ceftolozane/tazobactam, LOS: length of stay. * Rank of C/T initiation was defined by chronological order of start dates of all anti-pseudomonal Gram-negative antibacterials given for the index infection.

Overall, median (Q1; Q3) hospital length of stay (LOS) was 31.5 (16.0; 48.5) days in patients with CF (Table 2). When stratified by rank of C/T initiation, median hospital LOS was 23.0 (13.0; 45.0) days in patients who initiated C/T as first line treatment, 16.0 (11.5; 28.5) days when initiated as second treatment, 32.0 (15.0; 58.0) days when initiated as third treatment, 53.5 (28.5; 70.5) days when initiated as fourth treatment, 28.0 (21.0; 33.0) days when initiated as fifth treatment, and 46.5 (38.0; 82.0) days when initiated as sixth or later (Table 2, Figure 2). Median (Q1; Q3) post-C/T LOS was 2 (1.0; 13.0) days in patients with CF. When stratified by rank of C/T initiation, median LOS post-C/T treatment was 4.0 (1.0; 23.0) days in patients who initiated C/T as first line treatment, 1.0 (1.0; 6.0) day when initiated as second treatment, 3.0 (1.0; 24.0) days when initiated as third treatment, 2.5 (1.0; 30.5) days when initiated as fourth treatment, 1.0 (1.0; 4.0) day when initiated as fifth treatment and 3.0 (1.0; 31.0) days when initiated as sixth or later (Table 2). In total, four patients with CF remained hospitalized 30 days following C/T treatment (Table 2).

Overall, all-cause readmission within 30 days of C/T administration occurred in 10.9% of patients with CF (Table 2). When stratified by rank of C/T initiation, 30-day all-cause readmission was lowest in patients who initiated C/T as third, fourth or sixth or later treatment line (0.0%), followed by first line treatment (10.0%), second line treatment (12.5%) and fifth line treatment (44.4%) (Table 2). Infection-related readmission within 30 days of C/T administration was observed in 6.3% of patients with CF (Table 2). When stratified by rank of C/T initiation, 30-day infection-related readmission was lowest in patients who initiated C/T as second, third, fourth or sixth or more treatment line (0.0%), followed by first line treatment (10.0%) and fifth line treatment (33.3%; Table 2).

## 3. Discussion

In this subgroup analysis of SPECTRA, we describe outcomes and healthcare resource utilization among hospitalized adults with CF who received C/T for an index infection. Over half of the patients had MDR *P. aeruginosa*, underscoring the treatment complexity that can arise in hospitalized patients with CF. Although this analysis was not restricted to CF pulmonary exacerbations or respiratory infections, it remains clinically relevant because patients with CF frequently experience recurrent bacterial infections and are at increased risk of colonization and infection with antimicrobial-resistant Gram-negative pathogens. Accordingly, evaluating outcomes of C/T treatment in hospitalized patients with CF provides useful real-world context for management of serious Gram-negative infections in this high-risk population. Because index infection site/indication could not be consistently characterized in this subgroup, differences in outcomes by infection type cannot be assessed and are an important source of heterogeneity.

A total of 64 patients included in the SPECTRA study had CF, of which 53.1% (*n* = 34) had MDR strains of *P. aeruginosa*, highlighting the complexity of managing infections in hospitalized individuals with CF. However, the interpretation of these findings should consider differences in clinical context, including variation in infection type and severity, which were not consistently captured in this analysis. As such, the observed prevalence reflects the characteristics of the SPECTRA cohort and should be interpreted descriptively rather than as a direct comparison with studies focused on specific infection types [13]. Notably, the data collection period for this study occurred prior to that for SPECTRA, and therefore an increase in MDR *P. aeruginosa* strains is expected given the rising rates of MDR infections in recent years [13,14]. The evident increase in MDR *P. aeruginosa* highlights the urgent need for effective treatment against resistant infections and demonstrates the importance of antimicrobial stewardship programs and the ongoing need for further research into treatment protocols. This is especially important in the CF population where chronic infections, regular antibiotic treatment and MDR pathogens are common [15].

Clinical success was observed in 78.1% of hospitalized adults with CF treated with C/T in SPECTRA; however, this outcome reflects a composite, investigator-assessed measure applied across a heterogeneous set of infections. Differences in patient characteristics and clinical contexts may contribute to variation when comparing these findings with results from other populations. Accordingly, these results should be interpreted as descriptive of outcomes observed within this subgroup, rather than as evidence of treatment effect specific to any individual infection setting. Previously, clinical success for the total SPECTRA study cohort was 67.3% [12]. In addition, low all-cause (*n* = 9, 14.1%) and infection-related (*n* = 6, 9.4%) mortality rates were seen in patients with CF in this study. All-cause- and infection-related mortality rates were previously reported to be 21.2% and 7.6%, respectively [12]. However, this may be partly attributed to a wider variation in factors affecting health, such as comorbidities and pathogen strain, across the wider SPECTRA cohort.

Microbiological eradication was documented in 10.9% of patients; however, microbiological outcomes should be interpreted with caution in this population. Chronic colonization, particularly with *P. aeruginosa*, is common in CF, and follow-up cultures in routine clinical practice are not systematically collected. As a result, documented eradication may not fully reflect microbiological response and may underestimate treatment effects in this context.

Median LOS was lower in patients with CF (31.5 days) compared with the entire SPECTRA cohort (42.0 days) [12]. Furthermore, only four patients with CF remained hospitalized 30 days after the last day of C/T treatment, suggesting that C/T treatment may be associated with favorable outcomes in patients with CF. LOS findings in this analysis differ from those reported in prior studies, which may reflect differences in patient populations, underlying infection severity, and changes in antimicrobial resistance patterns over time. Additionally, earlier studies often focused on pulmonary exacerbations specifically, whereas the current analysis includes a broader range of infection types [16,17]. These contextual differences limit direct comparability across studies.

When stratified by rank of C/T initiation, rates of clinical success were comparable when C/T was initiated as the first to fifth treatment line (First line treatment *n* = 10, second line treatment *n* = 16, third line treatment *n* = 11, fourth line treatment *n* = 8, fifth line treatment *n* = 9, 72.7–100.0%) but decreased in patients treated with C/T as the sixth treatment or later (*n* = 4, 40.0%). These results suggest that C/T may be associated with favorable outcomes when initiated earlier in the treatment algorithm and also reflect the likely clinical complexity in treating patients who have failed five previous lines of therapy. An increase in mortality rates (infection-related and all-cause mortality) were also observed across later ranks of C/T initiation, with an infection-related mortality rate of 0.0% for patients treated with C/T as first or second line treatment, which increased to 30.0% (*n* = 3) for those treated with C/T as the sixth treatment or later. While variation in outcomes by treatment sequence may suggest differences associated with the timing of therapy initiation, these patterns should be interpreted cautiously. Patients receiving later-line treatment are likely to represent a more complex or treatment-refractory population, which may contribute to observed differences in outcomes. Small sample sizes within subgroups further limit the ability to draw definitive conclusions.

The results of this analysis of the SPECTRA study suggest that C/T may be associated with favorable outcomes in patients with CF, particularly in those with MDR *P. aeruginosa* infections, showing high clinical success and low mortality, readmission and LOS. The effectiveness of C/T can be further inferred from the CONDUCT study, a real-world study which identified high proportional use of C/T in patients with CF in France (25% of all prescriptions) [5]. These findings suggest that C/T may be used as a preferred treatment in patients with CF in real-world settings, likely due to its effectiveness in this population, increasing the confidence in the findings from the current SPECTRA study.

Given the high levels of respiratory infections, in particular MDR *P. aeruginosa*, and hypersensitivity to antimicrobials in patients with CF, there is an urgent need for effective treatment. As patients with CF are frequently treated with antibiotics, treatment that does not resolve the infection risks the further increase of the emergence of AMR and MDR pathogens. Further research is warranted to assess timing and rank of C/T initiation and comparative effectiveness, explore predictors of clinical success and microbial eradication in CF, and understand the stewardship implications in CF.

The analysis was not restricted to CF pulmonary exacerbations or respiratory infections, and index infection site/indication could not be consistently characterized for this CF subgroup. Therefore, results should be interpreted as descriptive outcomes among hospitalized patients with CF treated with C/T for heterogeneous serious infections, and comparisons with CF pulmonary exacerbation cohorts or the infection-site-specific literature are limited. The numbers of cases in the groups compared was very low and an adequate statistical analysis could not be performed. Microbiological outcomes reflect routine clinical practice (cultures not mandated and collected non-systematically) and are challenging to interpret in CF due to chronic colonization and biofilm-associated persistence. Clinical success was a composite, investigator-assessed outcome; given the heterogeneity of infection sites in SPECTRA and the presence of respiratory-specific components in the composite definition, the endpoint may not be equally applicable across all infection types.

## 4. Materials and Methods

SPECTRA was a multicenter, retrospective study which used medical records of adult inpatients treated with C/T for ≥48 h from January 2016 to November 2020. Real-world outcomes on C/T use were gathered from seven countries, including Australia, Austria, Germany, Italy, Mexico, Spain and the UK. Study design, population and core outcomes are defined in previous papers [10,11,12]. Patients were included if they were aged ≥18 years, had received C/T for ≥48 h, and had received their last C/T dose ≥ 30 days before data collection. This paper presents an analysis of a subgroup of patients with CF. Patients were excluded if they were participating in an interventional clinical trial for Gram-negative infections at the time of treatment.

The primary study objective was to characterize real-world treatment patterns, clinical outcomes and healthcare resource utilization (HCRU) in hospitalized adults receiving ≥48 h of treatment with C/T. A key secondary objective as presented in this manuscript was to analyze clinical outcomes and HCRU associated with C/T use in patients with CF.

The study was conducted in compliance with the International Society for Pharmacoepidemiology Guidelines for Good Pharmacoepidemiology Practices [18], the ethical principles arising from the Declaration of Helsinki [19], the European Union good pharmacovigilance practices [20], European and national laws with respect to data protection [21], and applicable local regulations.

### 4.1. Outcomes

Data were retrospectively collected from medical records covering up to 30 days after treatment or until death. Outcomes included investigator-assessed clinical success of C/T treatment during index infection, microbial eradication, in-hospital mortality (all-cause and infection-related), length of hospital stay following C/T administration, number of patients still hospitalized 30 days after last C/T dose, and all-cause and infection-related readmission rates. Data were stratified by rank of C/T treatment initiation line (first line treatment, second line treatment, third line treatment, fourth line treatment, fifth line treatment, sixth line treatment or more). The rank of C/T initiation was defined by the chronological order of start dates of all anti-pseudomonal Gram-negative antibacterials given for the index infection. The hospitalization during which the patient was first treated with ceftolozane/tazobactam was considered as the index hospitalization, and the infection for which the patient received C/T was considered as the index infection. The index date was the first date of administration of any antibiotic for any suspected Gram-negative infection that C/T was administered for. When two antibiotics were initiated on the same calendar date, rank was assigned using the antimicrobial spectrum hierarchy per the study statistical analysis plan (first line, second line, third line), with third line agents considered a later rank than second line, and second line a later rank than first line treatment.

Clinical success was defined as microbiological eradication, no Gram-negative therapy needed for a minimum of 48 h after administration of C/T (not including discharge antibiotics or de-escalation), no death due to Gram-negative infections, no additional treatment for exacerbation of respiratory infection within 28 days of stopping C/T, resolution of any exacerbations, or no need for re-operation for source control. Microbiology data were analyzed if collected within 14 days of C/T initiation and microbiological eradication was defined as negative culture at the site of index infection after C/T. Clinical success was assessed by the investigator for the index infection using the SPECTRA composite definition and was applied across infection types. Because SPECTRA enrolled hospitalized adults treated with C/T for heterogeneous index infections, the clinical success endpoint was captured as a single investigator-assessed variable in the eCRF (“Was the index infection considered a clinical success?” yes/no/unknown) and reflects routine clinical judgment using the SPECTRA composite framework. Some components of the composite definition are respiratory-specific (e.g., exacerbation of respiratory infection) and may be less applicable for non-respiratory infections; therefore, clinical success is interpreted here as an overall, investigator-assessed outcome for the index infection rather than as a CF pulmonary exacerbation-specific endpoint.

Patient readmission was recorded as not applicable if they were still hospitalized within 30 days of C/T administration. Hospital LOS was defined as the date of hospital discharge or death, minus the date of index hospitalization admission plus one, and was reported in days. For patients who remained hospitalized 30 days after the last day of C/T, the time from the last day of C/T to hospital discharge was imputed by 30 and the hospital LOS was then defined in days as the C/T stop date minus the date of index hospitalization admission plus 30 plus one. For patients who did not remain hospitalized 30 days past the last day of C/T for the index infection, post-C/T LOS was defined as date of hospital discharge or death minus the date of last dose of C/T plus one. Some patients may have been discharged from hospital before the last day of C/T for the index infection. In such a case, post-C/T LOS was 0. For patients who remained hospitalized 30 days after the last day of C/T, the time from the last day of C/T to hospital discharge was imputed by 30 and the post-C/T hospital LOS was then imputed by 31.

### 4.2. Statistical Analysis

Categorical variables were reported as counts and percentages, and continuous data were reported as mean and standard deviation (SD) or median and interquartile range (IQR). Where available, 95% confidence intervals (CIs) for proportions have been reported. No inferential statistics were performed on this dataset due to the small sample sizes, and therefore, descriptive statistics are reported only.

## 5. Conclusions

In this study, C/T demonstrated real-world effectiveness among patients with CF and MDR *P. aeruginosa* infections. In this population, clinical success was higher and all-cause in-hospital mortality, hospital LOS and readmission rates were lower than those observed in the total SPECTRA population. These observations may reflect differences in underlying patient complexity rather than the timing of therapy alone. We present results for the use of C/T in patients with CF from SPECTRA. Further research into C/T timing and rank, and into stewardship implications within this population may be warranted.

## Figures and Tables

**Table 1:** Clinical outcomes in CF patients treated with C/T, overall and by rank of C/T initiation.

	Rank of C/T Initiation *						
	First Line Treatment (*n* = 10)	Second Line Treatment (*n* = 16)	Third Line Treatment (*n* = 11)	Fourth Line Treatment (*n* = 8)	Fifth Line Treatment (*n* = 9)	Sixth Line Treatment or More (*n* = 10)	Total CF Population (*n* = 64)
Index infection considered as a clinical success (%)	8 (80.0)	16 (100)	8 (72.7)	6 (75.0)	8 (88.9)	4 (40.0)	50 (78.1)
95% CI	(44.4, 97.5)	(79.4, 100)	(39.0, 94.0)	(34.9, 96.8)	(51.8, 99.7)	(12.2, 73.8)	(66.0, 87.5)
Microbial eradication (%)	2 (20.0)	1 (6.3)	2 (18.2)	1 (12.5)	0	1 (10.0)	7 (10.9)
95% CI	(2.5, 55.6)	(0.2, 30.2)	(2.3, 51.8)	(0.3, 52.7)	-	(0.3, 44.5)	(4.5, 21.2)
All-cause in-hospital mortality (%)	0	0	2 (18.2)	2 (25.0)	1 (11.1)	4 (40.0)	9 (14.1)
95% CI	-	-	(2.3, 51.8)	(3.2, 65.1)	(0.3, 48.2)	(12.2, 73.8)	(6.6, 25.0)
Infection-related in-hospital mortality (%)	0	0	1 (9.1)	1 (12.5)	1 (11.1)	3 (30.0)	6 (9.4)
95% CI	-	-	(0.2, 41.3)	(0.3, 52.7)	(0.3, 48.2)	(6.7, 65.2)	(3.5, 19.3)

CF: cystic fibrosis, CI: confidence interval, C/T: ceftolozane/tazobactam. ‘*n*’ signifies the number of patients in each subgroup. * Rank of C/T initiation was defined by chronological order of start dates of all anti-pseudomonal Gram-negative antibacterials given for the index infection. ‘*n*’ refers to the number of patients included in each subgroup.

**Table 2:** HCRU associated with C/T use in patients with CF, overall and by rank of C/T initiation.

	Rank of C/T Initiation *						
	First Line Treatment (*n* = 10)	Second Line Treatment (*n* = 16)	Third Line Treatment (*n* = 11)	Fourth Line Treatment (*n* = 8)	Fifth Line Treatment (*n* = 9)	Sixth Line Treatment or More (*n* = 10)	Total CF Population (*n* = 64)
Number of patients who remain hospitalized 30 days after the last day of C/T	1	1	0	1	0	1	4
Hospital length of stay (days)							
Median (Q1; Q3)	23 (13.0; 45.0)	16 (11.5; 28.5)	32 (15.0; 58.0)	53.5 (28.5; 70.5)	28.0 (21.0; 33.0)	46.5 (38.0; 82.0)	31.5 (16.0; 48.5)
Min; Max	9; 102	8; 54	9; 127	17; 395	16; 94	31; 115	8; 395
Post-C/T length of stay (days)							
Median (Q1; Q3)	4 (1.0; 23.0)	1 (1.0; 6.0)	3 (1.0; 24.0)	2.5 (1.0; 30.5)	1 (1.0; 4.0)	3 (1.0; 31.0)	2 (1.0; 13.0)
Min; Max	1; 72	−5; 31	1; 67	1; 378	1; 44	1; 55	−5; 378
30-day all-causes readmission (%)	1 (10.0%)	2 (12.5%)	0	0	4 (44.4%)	0	7 (10.9%)
30-day infection-related readmission (%)	1 (10.0%)	0	0	0	3 (33.3%)	0	4 (6.3%)

CF: cystic fibrosis, C/T: ceftolozane/tazobactam, HCRU: health care resource utilization, SD: standard deviation. * Rank of C/T initiation was defined by chronological order of start dates of all anti-pseudomonal Gram-negative antibacterials given for the index infection. ‘*n*’ refers to the number of patients included in each subgroup.

## Data Availability

The full datasets generated and analyzed to inform the conclusions drawn within this manuscript during the current study are available from the corresponding author upon reasonable request.

## Supplementary Materials

The following supporting information can be downloaded at: https://www.mdpi.com/article/10.3390/antibiotics15080715/s1, Table S1. Number of patients with CF by country in the SPECTRA study; Table S2. Comorbidities in patients with CF in the SPECTRA study.
