# Supplementary material for: Clinical Outcomes and Healthcare Resource Utilization Results Among Hospitalized Adults with Cystic Fibrosis Treated with Ceftolozane/Tazobactam: A SPECTRA Real-World Subgroup Analysis
Source: Antibiotics (Basel). 2026 Jul 23;15(8):715. doi: 10.3390/antibiotics15080715 (PMC13509291; doi:10.3390/antibiotics15080715)
Supplement: Supplementary file 1 [file antibiotics-15-00715-s001.zip › antibiotics-4348079-supplementary.pdf]

Table S1. Number of patients with CF by country in the SPECTRA study.

	Australia	Austria	Germany	Italy	Mexico	Spain	UK	Total
**Total patient number**	59.0	12.0	29.0	57.0	59.0	313.0	88.0	617.0
CF patients (%)	12 (20.3)	0	4 (13.8)	1 (1.8)	1 (1.7)	11 (3.5)	35 (39.8)	64 (10.4)

CF: cystic fibrosis, UK: United Kingdom.

Table S2. Comorbidities in patients with CF in the SPECTRA study.

	CF patients
**Comorbidity (%)**	
Immunocompromised	36 (56.3)
Transplant	33 (51.6)
Critical care	25 (39.1)
Chronic pulmonary disease	45 (70.3)
Septic	7 (10.9)
Exacerbation of chronic respiratory infection	36 (56.3)
Pneumonia	15 (23.4)
Bone and joint infection	1 (1.6)
Wound/diabetic foot	0
HM	0
Solid tumor and HM	0
Sepsis/SIRS/Septic shock	15 (23.4)
**Renal impairment levels* (%)**	
End stage	0
Severe	3 (5.7)
Moderate	10 (15.6)
Mild	10 (15.6)
Normal	39 (60.9)
Augmented	1 (1.6)
**RRT (%)**	
First RRT during index hospitalization	4 (6.25)
First continuous RRT during index hospitalisation	3 (4.7)
First intermittent hemodialysis or peritoneal dialysis during index hospitalization	1 (1.6)
**BMI† (%)**	
Normal	32 (78.0)
Overweight	7 (17.1)
Obese	2 (4.9)

*Creatinine clearance (CrCl) was derived and categorized per the Statistical Analysis Plan. Renal impairment cut points used for the table are: end-stage <15 mL/min; severe 15–29 mL/min; moderate 30–59 mL/min; mild 60–89 mL/min; normal 90–149 mL/min; augmented ≥150 mL/min. †Patients with missing information on BMI are not presented in the table. For that reason, the sum of Ns in the BMI columns is inferior to the total N. BMI was derivable for 41/64 total patients. The remaining patients either had missing BMI data, or records were flagged as implausible during data cleaning and so were excluded. Percentages are calculated for patients where BMI information was available. BMI: body mass index, CF: cystic fibrosis, HM: hematologic malignancy, RRT: renal replacement therapy.
